# Supplementary material for: Iodine-Loaded Calcium Titanate for Bone Repair with Sustainable Antibacterial Activity Prepared by Solution and Heat Treatment
Source: Nanomaterials (Basel). 2021 Aug 26;11(9):2199. doi: 10.3390/nano11092199 (PMC8472594; doi:10.3390/nano11092199)
Supplement: Supplementary file 1 [file nanomaterials-11-02199-s001.zip › nanomaterials-1325856-supplementary.pdf]

Article

Iodine-Loaded Calcium Titanate for Bone Repair with Sustainable Antibacterial Activity Prepared by Solution and Heat Treatment

Seiji Yamaguchi [1,*], Phuc Thi Minh Le [1], Seine A. Shintani [1], Hiroaki Takadama [1], Morihiro Ito [1], Sara Ferraris [2] and Silvia Spriano [2]

[1] Department of Biomedical Sciences, College of Life and Health Sciences, Chubu University, 1200 Matsumoto, Kasugai, Aichi 487-8501, Japan; minhphuc@isc.chubu.ac.jp (P.T.M.L.); shintani@isc.chubu.ac.jp (S.A.S.); takadama@isc.chubu.ac.jp (H.T.); m-ito@isc.chubu.ac.jp (M.I.)

[2] Politecnico di Torino, Corso Duca degli Abruzzi 24, Corso Duca degli Abruzzi 24, 10129 Torino, Italy; sara.ferraris@polito.it (S.F.); d002307@polito.it (S.S.)

* Correspondence: sy-esi@isc.chubu.ac.jp; Tel.: +81-568-51-6420; Fax: +81-568-51-5370

A large amount or no amount of iodine incorporation was also observed when Ti was soaked in the ICl_3, or PVP-I solution following NaOH treatment, respectively. However, the critical scratch load of the treated Ti was low. The subsequent heat treatment increased the value to 34.7 mN but vanished the iodine from the metal surface due to low boiling point of iodine (~184 ºC).

Table S1. The results of quantitative analysis of XPS and scratch resistance measurement on Ti soaked in PVP-I or ICl_3 solution following NaOH treatment.

**Treatment**	**Element/mass %**				Critical scratch load /mN
	**O**	**Ti**	Ca	I	
NaOH-1000 ppm PVP-I 40 °C	50.0	49.9	0	0	8.0
NaOH-10 mM ICl_3 40 °C	40.0	49.8	0	10.2	5.5
NaOH-10 mM ICl_3 40 °C-heat	46.3	53.7	0	0	34.7

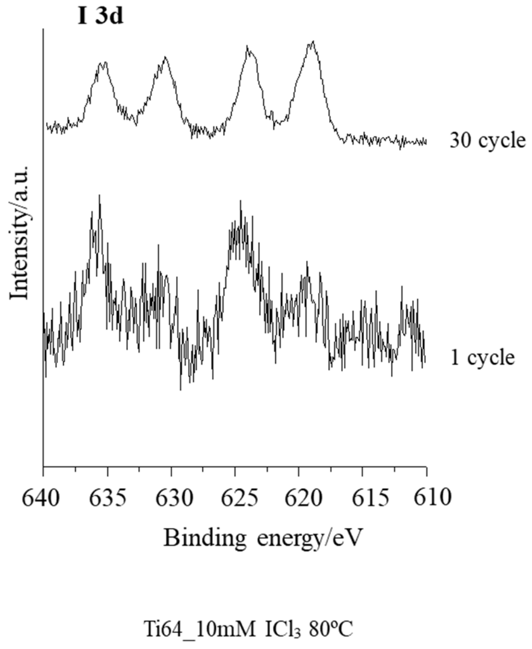

Figure S1. XPS profiles of I3d on Ti64_10mM ICl_3 80°C with measurement cycles of one and thirty.

After 1 cycle, strong peaks of 623 and 635 eV attributed to positively charged iodine ions were observed accompanied by weak peaks of 619 and 630 eV attributable to isolated/negatively charged iodine ions. The former decreased whereas the latter increased with increasing measurement cycles. This can be explained that the positively charged iodine ions transformed into isolated/negatively charged iodine ions by receiving electrons since the sample was exposed to electron beams during the measurement.
